# Metabolomics Assessment of Volume Overload-Induced Heart Failure and Oxidative Stress in the Kidney

**DOI:** 10.3390/metabo13111165

**Published:** 2023-11-20

**Authors:** Hsiang-Yu Tang, Jyh-En Huang, Ming-Tong Tsau, Chi-Jen Chang, Ying-Chang Tung, Gigin Lin, Mei-Ling Cheng

**Affiliations:** 1 Metabolomics Core Laboratory, Healthy Aging Research Center, Chang Gung University, Taoyuan City 33302, Taiwan; tangshyu@mail.cgu.edu.tw (H.-Y.T.); mttsau@mail.cgu.edu.tw (M.-T.T.); 2 Graduate Institute of Biomedical Sciences, College of Medicine, Chang Gung University, Taoyuan City 33302, Taiwan; andy_huang@waters.com; 3 Department of Cardiology, Linkou Chang Gung Memorial Hospital, Taoyuan City 33323, Taiwan; cchijen@cgmh.org.tw (C.-J.C.); n12374@cgmh.org.tw (Y.-C.T.); 4 School of Medicine, College of Medicine, Chang Gung University, Taoyuan City 33302, Taiwan; 5 Clinical Metabolomics Core Laboratory, Chang Gung Memorial Hospital, Taoyuan City 33323, Taiwan; giginlin@cgmh.org.tw; 6 Department of Medical Imaging and Intervention, Chang Gung Memorial Hospital at Linkou, Taoyuan City 33323, Taiwan; 7 Imaging Core Laboratory, Institute for Radiological Research, Chang Gung University, Taoyuan City 33323, Taiwan; 8 Department of Medical Imaging and Radiological Sciences, Chang Gung University, Taoyuan City 33302, Taiwan; 9 Department of Biomedical Sciences, College of Medicine, Chang Gung University, Taoyuan City 33302, Taiwan

**Keywords:** heart failure, volume overload, aortocaval fistula, cardiorenal syndrome, kidney, oxidative stress, uric acid, taurine

## Abstract

The incidence of heart failure (HF) is increasing and is associated with a poor prognosis. Moreover, HF often coexists with renal dysfunction and is associated with a worsened outcome. In many experimental studies on cardiac dysfunction, the function of other organs was either not addressed or did not show any decline. Until now, the exact mechanisms for initiating and sustaining this interaction are still unknown. The objective of this study is to use volume overload to induce cardiac hypertrophy and HF in aortocaval fistula (ACF) rat models, and to elucidate how volume overload affects metabolic changes in the kidney, even with normal renal function, in HF. The results showed the metabolic changes between control and ACF rats, including taurine metabolism; purine metabolism; glycine, serine, and threonine metabolism; glycerophospholipid metabolism; and histidine metabolism. Increasing the downstream purine metabolism from inosine to uric acid in the kidneys of ACF rats induced oxidative stress through xanthine oxidase. This result was consistent with HK-2 cells treated with xanthine and xanthine oxidase. Under oxidative stress, taurine accumulation was observed in ACF rats, indicating increased activity of the hypotaurine–taurine pathway as a defense mechanism against oxidative stress in the kidney. Another antioxidant, ascorbic acid 2-sulfate, showed lower levels in ACF rats, indicating that the kidneys experience elevated oxidative stress due to volume overload and HF. In summary, metabolic profiles are more sensitive than clinical parameters in reacting to damage to the kidney in HF.

## 1. Introduction

Heart failure (HF) is the ultimate outcome of various heart diseases and is a major cause of morbidity and mortality worldwide, with 64.3 million people estimated to be living with HF worldwide in 2017 [1]. The prevalence of HF increases with age when it occurs within the complex context of multimorbidity and geriatric syndromes [2]. In Asia, its prevalence seems similar to that in Western countries, ranging between 1% and 1.3% [3]. However, its prevalence in Taiwan is 6%, which is higher than in other countries in Asia [4]. Most patients with HF present with acute exacerbations of chronic heart failure (CHF) and often have evident comorbidities. The initial clinical syndrome in HF is hypertrophy, which is an important aspect of myocardial compensation aiming to improve myocardial contractility and maintain cardiac output [5]. Continued evolving hypertrophy eventually leads to the decompensation of heart function [6]. One of the most significant comorbidities that affects outcome and clinical management is renal failure or renal insufficiency [7]. This interaction between the heart and kidney, where decompensated HF can accelerate the deterioration of renal function, is known as cardiorenal syndrome type 2. The pathophysiology of cardiorenal syndrome involves various mechanisms, such as alterations in hemodynamics, the dysregulation of salt and fluid balance, endothelial dysfunction, inflammation, the activation of regulatory systems, and the sympathetic nervous system [8]. The most important factors are reduced renal perfusion and venous congestion, and worsening renal function is strongly related to increased mortality [9]. Even a small decrease in glomerular filtration rate is associated with increased mortality among patients with CHF [10]. There is emerging evidence that HF can also be considered as an inflammatory state that contributes to the gradual injury of renal cells, which may lead to chronic kidney damage [11]. There is a significant co-occurrence of HF and chronic kidney disease (CKD). Almost half of patients with HF have a degree of renal impairment, and HF is prevalent in 17–50% of patients with CKD [12]. A meta-analysis conducted on patients admitted to the hospital with HF has indicated that the presence of renal insufficiency is associated with a 1.5-fold increase in mortality compared to the control group. Furthermore, the mortality rate is found to be three times higher in patients with severe renal insufficiency [13]. Unfortunately, clinicians have been unable to accurately identify patients who have a poor prognosis following worsening renal function until now. Despite the considerable amount of knowledge gained on the interaction between the cardiovascular and renal systems in HF, questions and uncertainties remain. An experimental model of rats with aortocaval fistula (ACF) was established in our previous study to induce HF with volume overload [14,15,16]. This model resulted in severe cardiac hypertrophy and allowed us to characterize the metabolic derangements in cardiac energy metabolism [15]. According to our previous experiments [16], the results of a histological examination of the myocardium revealed that the size of cardiomyocytes underwent no significant increase after 8 weeks of ACF, but underwent significant enlargement after 16 weeks of ACF. These results indicated that the ACF rats remained at the compensatory phase at 8 weeks and progressed to decompensated HF at 16 weeks [17]. In a previous study, kidney tissue stained with hematoxylin–eosin showed tubular dilation and intratubular obstruction [16], indicating renal injury at 16 weeks after ACF. To clarify the impact of HF on kidney injury, 16-week ACF rats were used in this study. 

Metabolomics is the study of the complete profile of small-molecule metabolites within an organism, not only resulting from changes in the expression of genes, but also as a result of protein activity, including nutrition and drug therapies and environmental stimulation [18]. Myocardium switches from using fatty acid as an energy source to using glucose as an energy source in HF [19,20]. HF is understood to be a systemic, multi-organ syndrome with metabolic failure as the basic mechanism. We used liquid chromatography–mass spectrometry (LC-MS)-based metabolic profiling of plasma to evaluate the diagnostic and prognostic significance in a cohort of more than 400 patients with HF [21]. The findings demonstrate the efficacy of metabolomics in generating a strong prognostic and diagnostic metabolic model, regardless of brain natriuretic peptide (BNP) and other conventional risk factors. We also used LC-MS-based lipidomic profiling to identify 7-ketocholesterol in erythrocytes as an early marker of HF [22] and to assess metabolic status to predict the outcome of HF [23]. The implementation of metabolic profiles provides insight into various biological processes involved in the progression of HF and has the potential to improve the diagnosis and prognosis of cardiac diseases. Further information is required regarding the impact of HF on the kidneys due to the limited understanding of the significance of HF in the progression of kidney damage. Here, we use a metabolomics approach to elucidate the molecular mechanisms in the kidney tissue of ACF rat at 16 weeks underlying the progression of HF.

## 2. Materials and Methods

### 2.1. Experimental Animals

Male Sprague Dawley (SD) rats (aged 4 weeks) were purchased from BioLASCO Taiwan Co., Ltd. (Taipei, Taiwan). The SD rats were maintained in a climate-controlled facility on a 12 h light–dark cycle with ad libitum access to water and food. The animal experiments were performed with adherence to the basic standards of laboratory animal care according to the Guide for Care and Use of Laboratory Animals of the National Institutes, and were approved by the Institutional Animal Care and Use Committee of Chang Gung Memorial Hospital, Linkou, Taiwan (Approval number: 2009112701).

### 2.2. Establishment of the Rat Aortocaval Fistula (ACF) Model

The rat model of ACF was established following the previously described methods [14,15]. In brief, adult male SD rats (8–10-week-old) underwent an overnight fasting period (food was removed for 10 h) and were subsequently anesthetized using ketamine. A clamp was placed across the inferior vena cava (IVC) and aorta. An 18-gauge needle was used to puncture the lateral wall of the abdominal aorta. The needle was advanced to cross the opposite aortic wall toward the IVC and subsequently penetrate the adjacent wall of the IVC. The patency of the fistula was confirmed by a color change in the IVC. The sham-operated rats (control group) underwent a procedure involving the opening of the abdominal cavity without creating an ACF. All animal procedures were conducted in accordance with the guidelines of the committee on animal research at Chang Gung Memorial Hospital. After undergoing surgery, the SD rats were housed in a climate-controlled animal room, following a 12 h light–dark cycle with ad libitum access to water and food for a duration of 4 months.

### 2.3. Biochemical Analysis

After 4 months following the surgical procedure, blood samples were collected from the tail veins of the experimental rats after overnight fasting and added to a tube with ethylenediaminetetraacetic acid (EDTA, 4 mM). The whole blood was used for hematological analysis using an automated hematology analyzer (Sysmex, Milton Keynes, UK) in accordance with the user manual. The measured hematologic measurands included red blood cell (RBC) count, hemoglobin (HGB), hematocrit (HCT), mean corpuscular volume (MCV), mean corpuscular hemoglobin (MCH), mean corpuscular hemoglobin concentration (MCHC), white blood cell (WBC) count, platelet (PLT) count, red blood cell distribution width (RDW), red cell distribution width–coefficient of variation (RDW-CV), plateletcrit (PCT), platelet distribution width (PDW), and mean platelet volume (MPV). Plasma was isolated from whole blood via centrifugation at 1000× *g* for 10 min at 6 °C, and was then aliquoted and stored at −80 °C. Total cholesterol, triglyceride, phospholipid, creatinine, aspartate transaminase (AST), alanine transaminase (ALT), and B-type natriuretic peptide (BNP) levels were analyzed using Randox reagent kits (Randox Laboratories Ltd., Antrim, UK) and a rat BNP 32 ELISA kit (Abcam, Cambridge, UK) following the standard protocol in the user manual. 

### 2.4. Echocardiographic Evaluation of Cardiac Function

The rats received echocardiograms using ultrasound technology after 4 months of post-ACF as previously described [15]. Briefly, the rats were anesthetized with halothane inhalational anesthesia. Echocardiography was performed with multiple views using a 10 MHz ultrasound probe (Vivid System 5, GE, Boston, MA, USA), and calculations were recorded according to the American Society of Echocardiography guidelines [24]. M-mode echocardiography and 2-dimensional echocardiography images were obtained in the parasternal long- and short-axis views to determine the left ventricular structure and function. The thickness of the interventricular septum (IVS) and posterior wall (PW), left ventricular end-diastolic diameter (LVEDD), and left ventricular end-systolic diameter (LVESD) were determined at the tips of the papillary muscle. Left atrial (LA) diameters were measured in parasternal long-axis orientation. Fractional shortening (FS) was measured by M-mode according to the leading edge-to-leading edge convention in the short-axis view. 

### 2.5. Kidney Sample Preparation for Global Analysis of Hydrophilic Metabolites

Animal kidneys were quickly removed and snap-frozen in liquid nitrogen, and stored at −80 °C until further analysis. Kidney tissue was extracted using a modified method [25] and described as follows: frozen kidney tissue (about 100 mg) was ground together with liquid nitrogen in a cooled mortar. Homogenized samples were extracted with 4 mL of methanol and 0.8 mL of water and were transferred to glass tubes. Then, 4 mL of chloroform was added to the sample, and the sample was vortexed. Finally, 2 mL of water was added, and the sample was vortexed again and left on ice for 30 min. The final ratio of methanol/chloroform/water in the sample was 1:1:0.7 (*v*/*v*/*v*). Following centrifugation at 12,000× *g* for 30 min at 4 °C for phase separation, 5 mL of the aqueous metabolite-containing upper phase was transferred to another glass vial, and then, freeze-dried using a speed vacuum concentrator (Thermo Fisher, Milford, MA, USA). The dried sample was stored at -80°C before ultra-performance liquid chromatography coupled with time-of-flight mass spectrometry (UPLC TOF-MS) analysis. The dried sample was dissolved in 200 μL of 50% acetonitrile. Following centrifuging at 12,000× *g* for 30 min at 4 °C, the supernatant was transferred to a sample vial for analysis.

### 2.6. Determination of the Hydrophilic Profile of Metabolites in Kidney Tissue Using UPLC TOF-MS

For the separation of hydrophilic metabolites, an ACQUITY BEH Amide (2.1 mm × 150 mm, 1.7 μm, Waters, Milford, MA, USA) column was used. The column temperature was set at 45 °C, and the flow rate was set at 400 μL/min. Mobile phase A consisted of water with 0.1% formic acid, while mobile phase B consisted of acetonitrile with 0.1% formic acid. The initial LC gradient conditions were 99% B for 0.1 min, followed by a decrease to 30% B within 6.9 min. It was then brought back to 99% B for 0.2 min, and re-equilibrated for 2.8 min at 99% B. MS was performed using a Waters TOF-MS (SYNAPT G1 HDMS, Waters MS Technologies, Manchester, UK) operating in electrospray ionization (ESI)-positive and -negative ion modes. The desolvation gas was set at 800 L/h at a temperature of 500 °C; the source temperature was set at 120 °C. The capillary voltage was set at 2500 V in ESI-positive mode and 2000 V in ESI-negative mode, and cone voltage was set at 25 V, respectively. MS data were collected in centroid mode over a range of 50–1000 m/z at a rate of 0.1 scan/s. Sulfadimethoxine was used as the reference compound (an [M + H]^+^ ion at 311.0814 Da in ESI-positive mode; an [M + H]^−^ ion at 309.0658 Da in ESI-negative mode). Each sample was analyzed with four replicates to enhance the confidence in the quality. MassLynx V4.1 and MarkerLynx software V4.1 (Waters Corp., Milford, CN, USA) were used for feature identification and retention time correction, and were subjected to total ion normalization. Visualization matrices containing m/z paired with retention time, and peak area, were obtained. The metabolites were identified with accurate mass (<25 ppm) and the MS/MS data were compared with a standard database or online database (Supplementary Table S1).

### 2.7. Cell Culture and Cell Viability Determination

Human kidney-2 (HK-2) cells were cultured in Dulbecco’s modified Eagle medium (DMEM) (GIBCO Life Technologies, Bleiswijk, The Netherlands) with low glucose (5.5 mM), 10% fetal bovine serum (GIBCO Life Technologies, Bleiswijk, The Netherlands), 100 U/mL of penicillin, and 0.1 mg/mL of streptomycin in a humidified atmosphere containing 5% CO_2_ at 37 °C. For the oxidative status assay, 5 × 10^4^ cells were cultured in 12-well plates at 37 °C. After a 24 h culture, the DMEM was changed to serum-free DMEM and pre-treated with or without 5 mM of hypotaurine or taurine for 2 h. Then, the cells were incubated in different doses of H_2_O_2_ (0.5 mM, 1 mM, and 1.5 mM) or xanthine (1 mM, 1.5 mM, and 2 mM) with 5 mU/mL xanthine oxidase (XO) for 24 h. At the end of the treatments, the cells were fixed with a 3.7% formaldehyde solution, and then, stained with 1 mL of 10 mg/mL Hoechst 33342 for 2 h. The illumination of fluorescence was detected using an INCell Analyzer 1000 (GE Healthcare Bio-Sciences, Chicago, IL, USA).

### 2.8. Statistical Analysis

For multivariate data analysis, a visualization model including unsupervised principal component analysis (PCA) and a supervised orthogonal partial least squares discriminant analysis (OPLS-DA) model were performed using the SIMCA-P software (version 13.0, Umetrics AB, Umea, Sweden). The PCA score plots were visualized with the first principal component t [1] and the second principal component t [2]. The model validity was evaluated with the parameters of R2X (cum) and Q2 (cum). R2X is the total variation explained in the data and Q2 is the cross-validated explained variation. The reliability of the models increases with R2X and Q2 approaching 1. The significantly different metabolites were determined using a two-sample *t*-test (*p* < 0.05) and variable importance in the projection (VIP) values (≥1.0). VIP scores indicate which variables contributed most to the group separation observed in the OPLS-DA scores plot. Those significantly changed metabolites were searched from the HMDB (http://www.hmdb.ca/, accessed on 8 October 2023) or METLIN (https://metlin.scripps.edu/index.php, accessed on 8 October 2023) databases and were identified using the same parameters in positive and negative ESI mode using the reference standards in our home-made database and public databases with a 10 ppm tolerance. The most abundant metabolite, which appeared in both positive and negative modes or with different adducts, was selected for display. Metaboanalyst 5 software (https://www.metaboanalyst.ca/, accessed on 8 October 2023) was used for statistical analysis and pathway analysis. For all experiments, the results are reported as the mean ± SD (SEM). The statistical significance was determined using a Mann–Whitney U test for comparing between groups. Asterisks represent the follosing: * *p* < 0.05, ** *p* < 0.01, and *** *p* < 0.001.

## 3. Results

### 3.1. Hemodynamic Characteristics and Cardiac Structure

**Figure 1:**
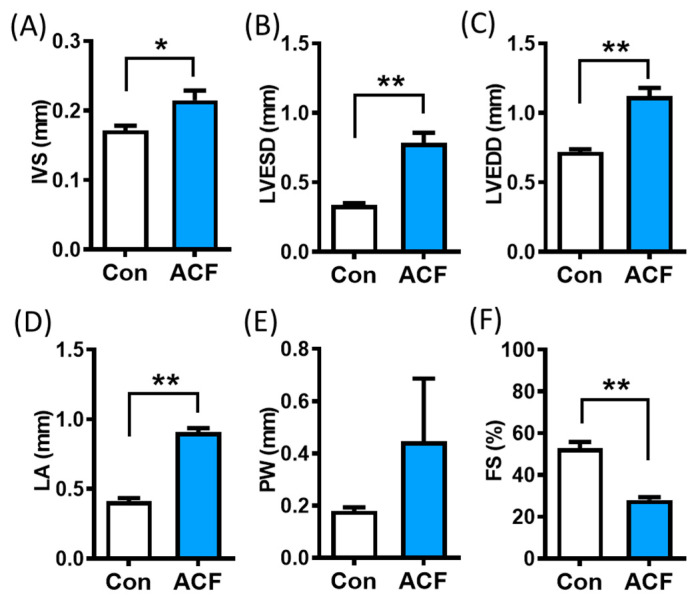
Evaluation of the aortocaval fistula (ACF) model using different echocardiographic parameters. Changes in echocardiography of the interventricular septum thickness (IVS) (**A**), left ventricular end-systolic dimension (LVESD) (**B**), left ventricular end-diastolic dimension (LVEDD) (**C**), left atrium (LA) dimension (**D**), posterior wall thickness (PW) (**E**), and fractional shortening (FS) (**F**) in ACF group (*n* = 6) and control group (*n* = 6) at 4 months after surgery. Data are reported as mean ± SEM. Differences between two groups were determined via Mann–Whitney U test. * *p* < 0.05; ** *p* < 0.01.

Hemodynamic and laboratory analyses were performed at 4 months post-ACF (Table 1). HCT and HGB were significantly lower in the ACF group compared to the control group. Though the RBC level was lower in ACF rats, the difference was not statistically significant. BNP level and AST activity were significantly higher in the ACF group. The echocardiographic data showed that the IVS thickness of the left ventricle, LVESD, LVEDD, and LA dimension significantly increased in the ACF group compared to the control group (Figure 1A–D). Additionally, FS was significantly reduced in the ACF group (Figure 1F). However, the thickness of PW did not change significantly between the ACF and control groups (Figure 1E). These results reveal that SD rats subjected to ACF exhibited decompensated heart failure.

### 3.2. Metabolomics Analysis of Kidney Metabolites in Control and ACF Rats

**Figure 2:**
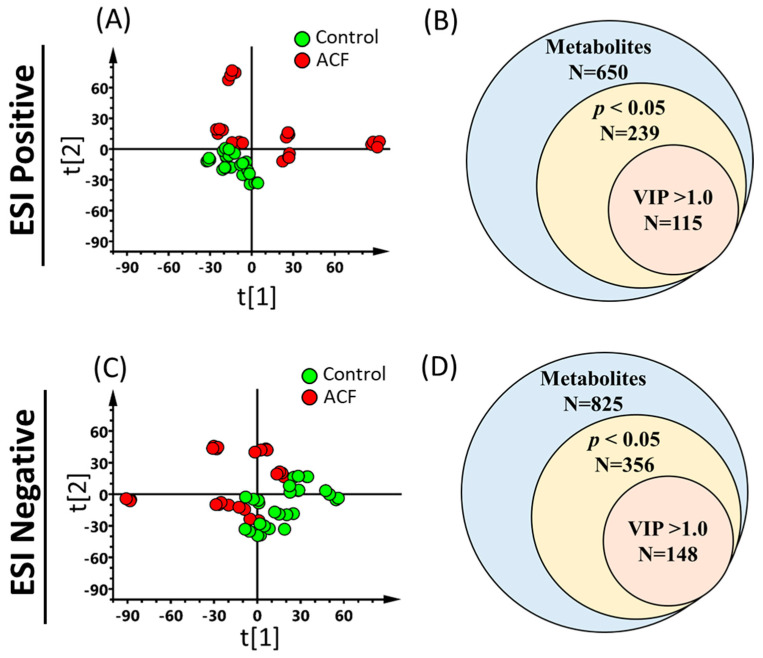
Untargeted metabolites were found to be altered between the aortocaval fistula (ACF) and control groups. Kidney tissue from ACF (*n* = 6) and control (*n* = 7) rats was extracted for UPLC-TOFMS analysis using electrospray ionization (ESI)-positive and ESI-negative modes. Unsupervised score plots of principal component analysis (PCA) (**A**,**C**) show considerable separation between the ACF (red circle) and control (green circle) groups (R2X = 0.759, Q2 = 0.46 in positive mode; R2X = 0.736, Q2 = 0.459 in negative mode). Each animal was analyzed with four replicates to enhance the confidence in the quality. Metabolites with significant changes and with more contributions in positive (N = 115) (**B**) and negative (N = 148) (**D**) modes were selected based on *p* < 0.05 (ACF versus control) and variable importance in projection (VIP) scores >1 in orthogonal partial least squares discriminant analysis (OPLS-DA) model, respectively.

Uremic toxins are involved in kidney dysfunction. However, the levels of p-cresyl sulfate and indoxyl sulfate did not significantly change between the control and ACF groups (Supplementary Figure S1). To investigate whether ACF causes metabolic changes in the kidney, we conducted untargeted metabolomics to evaluate the metabolic profiling in rat kidneys. After peak picking, alignment, and normalization to total ion abundance, there were 650 metabolites in positive mode and 825 metabolites in negative mode (Figure 2B,D). The unsupervised multivariate analysis with visualized PCA showed clear group separation between the control and ACF groups in both ESI-positive mode (Figure 2A) and ESI-negative mode (Figure 2C) based on the 650 metabolites in positive mode and 825 metabolites in negative mode. To determine the metabolic differences between the ACF and control animals, a two-sample *t*-test was applied to all metabolites. In total, 239 metabolites and 356 metabolites changed significantly (*p* < 0.05) (Figure 2B,D). Those metabolites that significantly changed were selected again based on VIP ≥ 1.0, which made a significant contribution to the OPLS-DA model (Supplementary Figure S2). A total of 115 and 148 features exhibited significant differences and contributions in the kidney tissue of ACF rats compared to the control group in positive and negative modes, respectively (Figure 2B,D). Table 2 shows the significantly changed metabolites in positive and negative mode between the ACF and control groups.

**Figure 3:**
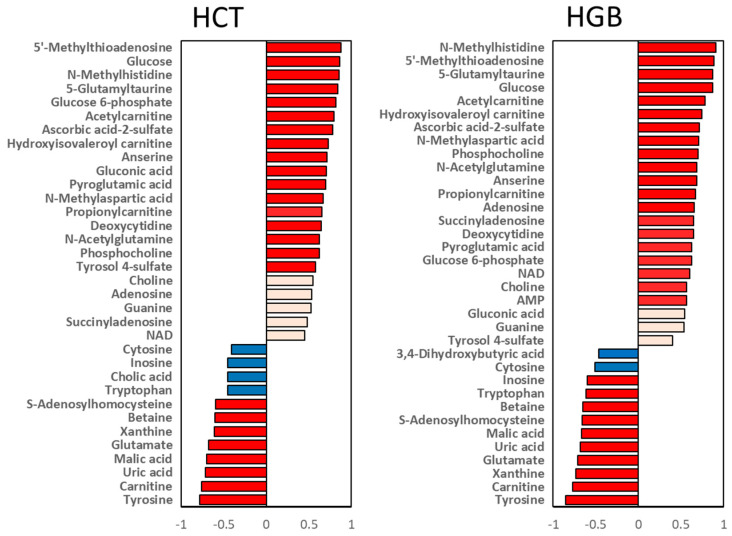
Correlation analysis between metabolites and biochemical value. Significantly changed metabolites were correlated to hematocrit (HCT) and hemoglobin (HGB). The orange and blue indicate positive and negative correlations, and the red color indicates a strong correlation (*p* < 0.05) with significance. NAD, nicotinamide adenine dinucleotide; AMP, adenosine monophosphate.

To elucidate the correlation between changes in kidney metabolites and HF with anemia, we correlated HCT and HGB with the metabolites that showed significant changes in the ACF group (Figure 3). There are 17 metabolites and 8 metabolites that are significantly positively and negatively correlated with HCT, respectively, and 20 metabolites and 10 metabolites that are significantly positively and negatively correlated with HGB, respectively. These data indicate that changes in metabolites in kidney tissue are correlated with HCT and HGB levels in the blood.

**Figure 4:**
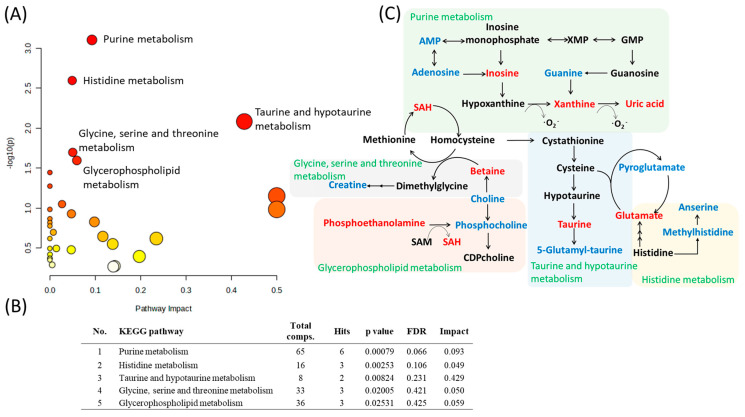
Pathway analysis shows altered metabolic pathways in kidneys with heart failure in aortocaval fistula (ACF) rats. (**A**) Metabolites that displayed significantly different abundance between the ACF and control group were subjected to pathway enrichment analysis using the MetaboAnalyst 5 software package. The matched pathways are arranged by *p* values on the *y*-axis. The pathway impact values (from pathway topology analysis) are plotted on the *x*-axis. The colors of the nodes are based on their *p* values (darker colors indicate more significant changes in metabolites in the corresponding pathway) and the size of the nodes is based on their pathway impact values, with a big size for large degree values. (**B**) The top five pathways that arise with high impact and low p values are indicated in table format. (**C**) Interaction of those significantly changed metabolites in pathways. Metabolites marked in red ink refer to those significantly increased in ACF tissue. Metabolites marked in blue ink refer to those significantly decreased in ACF tissue. Metabolites marked in black indicate no significant difference between the ACF and control groups or no measurement.

To identify dysregulated pathways in the kidney post-ACF, we conducted pathway analysis on the metabolites that showed significant differences in abundance between the ACF and control rats. The pathway analysis was performed on the combined positive- and negative-mode data. The results showed that five different pathways were significantly affected in the kidneys of the ACF group, including purine metabolism (*p* < 0.001); histidine metabolism (*p* < 0.01); taurine and hypotaurine metabolism (*p* < 0.01); glycine, serine, and threonine metabolism (*p* < 0.05); and glycerophospholipid metabolism (*p* < 0.05) (Figure 4A,B). The details of the interaction between each pathway are depicted in Figure 4C.

### 3.3. Accumulation of Xanthine and Uric Acid in Kidney Tissue Indicates a Higher Oxidative Stress Status in Kidney Cells

**Figure 5:**
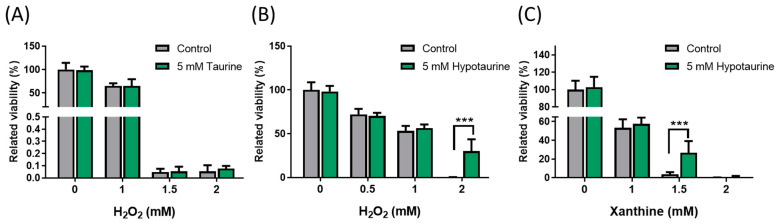
Cell viability in H_2_O_2_ or xanthine treatment following taurine or hypotaurine pretreatment. HK-2 cells were pretreated with 5 mM of taurine (**A**) and 5 mM of hypotaurine (**B**,**C**) for 2 h, and then, exposed to different doses of H_2_O_2_ (**A**,**B**) or various doses of xanthine with 5 mU/mL xanthine oxidase (**C**) for 24 h. The viabilities of the treated cells were determined using an automated cell counter and expressed as percentages relative to the control cells without H_2_O_2_ or xanthine treatment. Data are presented as means ± SD, *n* = 3; *** *p* < 0.001 for hypotaurine-pretreated cells vs. control cells.

Metabolomics analysis of the kidneys from ACF rats revealed higher levels of xanthine, uric acid, and taurine in kidney tissue than in control rats (Figure 4C). These elevated levels of xanthine and uric acid are typically caused by the activity of the enzyme XO. A higher level of taurine is the end product of the oxidation of hypotaurine, and taurine also functions as a potent free radical scavenger [26]. To investigate the role of taurine and hypotaurine in kidney cells during oxidative damage caused by xanthine and XO, we examined the viability of HK-2 cells that were pre-treated with 5 mM taurine (Figure 5A) or 5 mM hypotaurine (Figure 5B) for 2 h. Subsequently, the cells were exposed to different doses of H_2_O_2_ or varying doses of xanthine and 5 mU/mLXO for 24 h (Figure 5C). H_2_O_2_ and xanthine caused a dose-dependent reduction in the viability of HK-2 cells. Pretreatment with hypotaurine, but not taurine, was able to rescue the HK-2 cells from oxidative damage (Figure 5B,C).

## 4. Discussion

In the present study, our data demonstrate that ACF rats exhibit symptoms of HF due to abnormal heart function, including high values of IVS, LVESD, LVEDD, and LA dimension, low values of FS, as well as higher levels of BNP after four months of ACF (Figure 1 and Table 1). Lower HGB and RBC has been reported as factor contributing to anemia. Hemodilution is common in CHF patients. Lower HCT may result from volume overload in CHF, which tends to have worse outcomes than those of CHF patients with true anemia [27], suggesting that volume overload may be an important mechanism contributing to the poor outcome in anemic HF patients. Anemia and iron deficiency are two significant comorbidities in patients with HF, and they are linked to a poor clinical status and worse outcomes [28]. Anemia is caused by multifactorial factors such as chronic inflammation, renal dysfunction, and erythropoietin resistance [29]. Erythropoietin is primarily produced within the renal cortex and outer medulla by peritubular fibroblasts in response to hypoxia. A decline in cardiac function leads to worsening kidney function in cardiorenal syndrome type 2 [30], and renal dysfunction or CKD could reduce erythropoietin production [31,32]. A histological analysis of kidneys in our previous study revealed renal injury after 4 months of ACF [16], but the plasma levels of creatinine and uremic toxins did not change significantly in ACF rats. The BNP level was elevated in patients with renal dysfunction [33,34]. However, BNP levels can be falsely low in patients with obesity and advanced HF due to myocardial fibrosis.

Uremic toxins, such as indoxyl sulfate (IS) and p-cresol sulfate (PCS), accumulate in CKD patients, leading to a decline in renal function [35] and impairing NO synthesis in endothelial cells [36]. The roles of IS and PCS may also extend to participating in the complex interrelationship between the heart and the kidney. While the levels of IS and PCS did not change significantly in the kidneys and plasma of ACF rats in the present study, this indicates that more than 4 months are needed to induce kidney damage. Based on the above results, the kidneys may not suffer severe injury in ACF rats after 4 months of volume overload. The damage mechanism in the kidneys in ACF-induced HF may be different from that in typical cardiorenal syndrome, as demonstrated in a model developed by Van den Eynde J. et al. [37], which involves a combination of five-sixths partial nephrectomy and ACF to induce cardiorenal syndrome. This model resulted in higher levels of creatinine and indoxyl sulfate compared to a sham group after 3 weeks.

In the present metabolic study, ACF kidneys demonstrated that 36.8% and 43% of metabolites were affected in the positive and negative modes after 4 months of volume overload. This indicates that metabolic profiling is more sensitive in assessing the condition of the kidney compared to uremic toxins. Marked abnormalities in kidney metabolism include purine metabolism, taurine metabolism, and histidine metabolism. The purine metabolic pathway includes the conversion of hypoxanthine to xanthine and xanthine to uric acid by XO [38]. This process generates reactive oxygen species, such as superoxide radicals and hydrogen peroxide, which can cause cellular damage. The potential benefit of XO inhibition has been studied [39,40,41]. Our HK-2 cell experiment showed that treating cells with various doses of xanthine has the same effect as H_2_O_2_ treatment, leading to cell death (Figure 5). Several lines of evidence indicate that a high concentration of uric acid in the blood can lead to renal interstitial fibrosis and accelerate the progression of CKD [42], and disturbed uric acid metabolism may mediate cardiorenal syndrome [43]. A potential explanation may be that uric acid clearance is impaired in CKD, resulting in the elevation of plasma uric acid levels. Uric acid levels in the blood increase even in the early stages of kidney disease, making it a potential sensitive indicator of impaired kidney function.

Reviews have mentioned that taurine participates in various important biological functions in the kidney, such as ion transport, osmoregulation during the stress response, antioxidant activity, and neurotransmission [44]. The accumulation of taurine in our study may also serve similar functions to the kidney. Though the liver is the primary site of endogenous taurine synthesis, smaller amounts are also produced in peripheral organs, including the kidneys [45]. In addition to obtaining taurine from the diet, most taurine can also be synthesized from hypotaurine by flavin-containing monooxygenase (FMO1) in the livers of mice [46]. However, this synthesis does not occur in the livers of adult humans. Thus, other extrahepatic tissues, such as the kidney, brain, and heart, also express FMO1. This suggests that the sharing of intermediates between tissues may play a significant role in humans [47]. Taurine has been shown to act as an antioxidant in various studies by enhancing both enzymatic and nonenzymatic antioxidant systems. However, the specific mechanisms underlying its antioxidant properties remain unknown. The well-known antioxidant function of taurine is its capability to neutralize hypochlorous acid, forming taurine chloramine. This compound aids in the systemic scavenging of ROS [48]. Taurine chloramine has been shown to downregulate proinflammatory mediators such as chemokines and cytokines, and to upregulate Nrf2-dependent cytoprotective gene expression [49]. They are prevalent in the early phase of inflammation in the tubules [50]. The kidneys of transgenic mice that overexpressed the human taurine transporter had higher levels of intracellular taurine and less necrosis and apoptosis compared to control mice during renal damage caused by cisplatin [51]. An interesting corollary is that taurine supplementation might also lead to an increase in the levels of its precursors, such as the antioxidants hypotaurine and cysteine [47]. Hypotaurine is an organic osmolyte that acts as an antioxidant to scavenge ROS. In the present study, hypotaurine was found to be more effective than taurine in protecting HK-2 cells from oxidative damage. The protective role of taurine in ACF kidneys may not be directly related to redox reactions. However, it may induce the expression of other genes and proteins related to antioxidants to defend against oxidative damage.

Ascorbic acid 2-sulfate is one of several derivatives of 2-O-substituted ascorbic acid that have been discovered to possess radical-scavenging activity. Unlike other derivatives, it does not require enzymatic conversion to ascorbic acid and can directly react with radicals, though without ascorbic acid as an intermediate [52]. Our study also found a decreased level of ascorbic acid 2-sulfate in the kidneys of ACF rats. Takebayashi et al. demonstrated that the quenching of free radicals by ascorbic acid 2-sulfate was comparable to or greater than that of ascorbic acid over a prolonged period [52]. In the current study, it was found that ascorbic acid 2-sulfate has an impact on a wide range of diseases, including cardiomyopathy [53]. This compound may play a protective role in the kidneys of ACF rats.

Taurine is preferentially localized in the mitochondria because of the presence of a taurine transporter system across the mitochondrial membranes. This system stabilizes mitochondrial oxidation and metabolic function by acting as a matrix buffer [54]. In our results, we observed a decrease in the levels of glucose, hexose 6-phosphate, and NAD in the glycolysis pathway, as well as an increase in the levels of glutamate and malate in the tricarboxylic acid cycle (Table 2). These findings suggest that the energy requirement in the kidneys of ACF rats is associated with higher levels of taurine. Furthermore, taurine mediates energy generation and fatty acid oxidation [55] by regulating essential mitochondrial genes such as *Ucp1*, *Ucp2*, and PR domain-containing 16 [56]. The function of taurine in stimulating fatty acid oxidation is mediated by several important enzymes, including carnitine palmitoyltransferase 1-α, lipoprotein lipase, peroxisome proliferator-activated receptor-γ, aconitase 1, and aconitase 2 [56]. Our results also show that acetyl carnitine, propionyl carnitine, and hydroxyisovaleroyl carnitine are significantly lower in ACF rats, indicating an increase in the turnover of β-oxidation in mitochondria.

## 5. Conclusions

These findings highlight that volume overload-induced decompensated heart failure causes metabolic disturbances in the kidney, providing insight into the pathogenesis of cardiorenal dysfunction. We demonstrated associations between HF and altered kidney metabolism. In ACF rats, HF induces oxidative stress in the kidney, which is the primary burden and a potential factor contributing to the occurrence of cardiorenal syndrome type 2. Future studies are needed to explore the details of the underlying mechanism of ACF-induced abnormalities in kidney metabolites.

## Figures and Tables

**Table 1:** Blood hematologic and biochemistry data of control and ACF rats after surgery for 4 months.

	Control (*n* = 7)	ACF (*n* = 6)	*p*-Value
Weight (g)	514.29 ± 52.16	579.17 ± 73.65	0.133
Hematologic data			
WBC (10^3^/μL)	14.85 ± 5.04	16.15 ± 5.06	0.366
RBC (10^6^/μL)	10.59 ± 0.55	9.18 ± 1.41	0.073
HGB (g/dL)	16.91 ± 0.62	14.65 ± 1.97 *	0.017
HCT (%)	52.51 ± 1.33	46.10 ± 4.93 **	0.008
MCV (fL)	49.73 ± 2.97	50.62 ± 4.02	0.628
MCH (pg/cell)	16.00 ± 0.78	16.00 ± 0.73	0.943
MCHC (g/dL)	32.23 ± 1.38	31.70 ± 1.33	0.471
RDW (fL)	29.36 ± 0.60	30.57 ± 3.33	0.836
RDW-CV (%)	20.79 ± 1.47	20.17 ± 1.43	0.668
PLT (10^3^/μL)	959.86 ± 215.68	741.33 ± 221.89	0.100
PCT (%)	0.71 ± 0.18	0.54 ± 0.15	0.101
PDW (fL)	8.16 ± 0.24	8.03 ± 0.68	0.564
MPV (fL)	7.31 ± 0.34	7.30 ± 0.38	0.942
Biochemistry data			
Glucose (mg/dL)	87.86 ± 9.35	89.67 ± 15.37	1.000
Creatinine (mg/dL)	1.09 ± 0.31	1.13 ± 0.14	0.775
BNP (ng/mL)	0.49 ± 0.28	1.10 ± 0.62 *	0.036
ALT (U/L)	16.68 ± 6.16	17.88 ± 4.30	0.945
AST (U/L)	54.11 ± 12.75	76.55 ± 19.29 *	0.022
Phospholipid (mg/dL)	93.52 ± 8.86	97.73 ± 8.90	0.445
Cholesterol (mg/dL)	167.20 ± 10.84	167.06 ± 15.79	0.945
Triglyceride (mg/dL)	70.68 ± 16.78	77.33 ± 16.00	0.534

Data are presented as mean ± SD. Variables were analyzed via Mann–Whitney U test between control (*n* = 7) and ACF (*n* = 6) rats. WBC, white blood cell; RBC, red blood cell; HGB, hemoglobin; HCT, hematocrit; MCV, mean corpuscular volume; MCH, mean corpuscular hemoglobin; MCHC, mean corpuscular hemoglobin; RDW, red blood cell distribution width; RDW-CV, RDW–coefficient of variation; PLT, platelet; PCT, plateletcrit; PDW, platelet distribution width; MPV, mean platelet volume; BNP, B-type natriuretic peptide; AST, aspartate transaminase; ALT, alanine transaminase. * *p* < 0.05; ** *p* < 0.01.

**Table 2:** List of identified metabolites that were significantly changed between control and aortocaval fistula (ACF) groups.

No.	Metabolites	Control	ACF	VIP	*p* Value
1	Carnitine	517.03 ± 45.87	606.07 ± 101.21	4.59	1.15 × 10^−4^
2	Acetylcarnitine	72.99 ± 13.65	28.75 ± 20.74	4.04	2.39 × 10^−12^
3	Phosphocholine	283.01 ± 28.89	240.81 ± 38.19	3.25	3.68 × 10^−5^
4	Adenosine	34.62 ± 16.26	11.34 ± 4.55	2.74	1.31 × 10^−8^
5	Betaine	143.77 ± 13.45	178.44 ± 61.52	2.48	5.43 × 10^−3^
6	Taurine	209.68 ± 17.40	236.60 ± 31.51	2.46	3.00 × 10^−4^
7	NAD	113.83 ± 30.33	87.52 ± 16.21	2.41	3.87 × 10^−4^
8	Creatine	71.38 ± 18.67	48.59 ± 25.99	2.21	5.94 × 10^−4^
9	Guanine	57.76 ± 13.18	40.64 ± 12.72	2.11	1.79 × 10^−5^
10	Propionylcarnitine	15.17 ± 6.40	3.17 ± 3.77	2.05	1.36 × 10^−1^
11	Choline	78.12 ± 35.95	50.35 ± 46.63	2.05	1.91 × 10^−2^
12	Hydroxyisovaleroyl carnitine	31.17 ± 5.16	20.01 ± 9.03	1.79	1.02 × 10^−6^
13	Anserine	23.66 ± 7.91	12.19 ± 8.25	1.77	5.12 × 10^−6^
14	Deoxycytidine	6.84 ± 3.09	0.62 ± 1.16	1.52	1.69 × 10^−12^
15	N-Methyl histidine	26.77 ± 3.12	19.86 ± 6.61	1.36	9.22 × 10^−6^
16	N-Methyl aspartic acid	11.27 ± 5.95	4.98 ± 3.70	1.25	4.24 × 10^−5^
17	Tryptophan	21.16 ± 6.38	28.66 ± 8.91	1.25	9.05 × 10^−4^
18	Cytosine	44.11 ± 7.58	52.09 ± 11.49	1.20	4.29 × 10^−3^
19	5′-Methylthioadenosine	11.03 ± 1.37	7.70 ± 1.57	1.08	9.21 × 10^−11^
20	Inosine	949.86 ± 93.69	1021.77 ± 95.09	3.60	8.49 × 10^−3^
21	AMP	191.35 ± 97.41	127.16 ± 78.60	3.32	1.27 × 10^−2^
22	Pyroglutamic acid	127.33 ± 23.56	100.05 ± 21.89	2.66	7.91 × 10^−5^
23	Xanthine	93.10 ± 24.54	128.83 ± 59.43	2.61	5.41 × 10^−3^
24	Glucuronolactone	95.91 ± 46.76	61.38 ± 40.11	2.53	6.65 × 10^−3^
25	Tyrosine	20.93 ± 4.00	34.82 ± 9.55	2.21	5.60 × 10^−9^
26	Succinyladenosine	49.98 ± 17.85	33.01 ± 10.59	2.06	1.62 × 10^−4^
27	Malic acid	56.50 ± 9.38	72.07 ± 15.28	2.04	4.11 × 10^−5^
28	Gluconic acid	32.49 ± 11.13	20.62 ± 9.06	1.74	1.20 × 10^−4^
29	Glutamate	106.18 ± 12.36	119.18 ± 17.13	1.63	2.60 × 10^−3^
30	Uric acid	24.67 ± 3.15	34.37 ± 11.54	1.58	8.60 × 10^−5^
31	Glucose 6-phosphate	17.48 ± 4.50	10.55 ± 5.18	1.43	4.16 × 10^−6^
32	Ascorbic acid-2-sulfate	27.11 ± 6.49	18.16 ± 11.54	1.41	9.53 × 10^−4^
33	Cholic acid	6.32 ± 7.45	14.00 ± 8.03	1.32	7.85 × 10^−4^
34	Glucose	9.75 ± 1.83	4.97 ± 2.89	1.31	2.63 × 10^−9^
35	S-Adenosylhomocysteine	6.89 ± 2.24	11.31 ± 3.42	1.17	9.50 × 10^−7^
36	3,4-Dihydroxybutyric acid	2.14 ± 2.94	6.39 ± 2.33	1.15	6.30 × 10^−7^
37	5-Glutamyl-taurine	38.93 ± 4.00	32.95 ± 8.58	1.12	1.80 × 10^−3^
38	Phosphoethanolamine	25.59 ± 7.16	31.09 ± 3.73	1.09	1.39 × 10^−3^
39	N-Acetylglutamine	4.89 ± 2.20	1.71 ± 1.40	1.02	1.39 × 10^−7^
40	Tyrosol 4-sulfate	7.35 ± 3.96	3.37 ± 2.61	1.01	1.13 × 10^−4^

Data are presented as mean ± SD. Variables were analyzed via two-sample t-test between control (*n* = 7) and ACF (*n* = 6) groups. ACF, aortocaval fistula; NAD, nicotinamide adenine dinucleotide; AMP, adenosine monophosphate.

## Data Availability

The data presented in this study are available within the article.

## Supplementary Materials

The following supporting information can be downloaded at: https://www.mdpi.com/article/10.3390/metabo13111165/s1, Figure S1: Levels of p-cresol sulfate and indoxyl sulfate in plasma and kidney. Figure S2: Score plot from the multivariate OPLS-DA model in positive and negative mode. Table S1: Illustration of the identified metabolites from ESI-positive or -negative mode using the laboratory standard database or the online Human Metabolome Database. Supplementary method describes the detail of LC-MS method for p-cresol sulfate and indoxyl sulfate quantification.
