# Supplementary material for: Metabolomics Assessment of Volume Overload-Induced Heart Failure and Oxidative Stress in the Kidney
Source: Metabolites. 2023 Nov 20;13(11):1165. doi: 10.3390/metabo13111165 (PMC10672757; doi:10.3390/metabo13111165)
Supplement: Supplementary file 1 [file metabolites-13-01165-s001.zip › metabolites-2678034-supplementary.pdf]

Supplementary Information

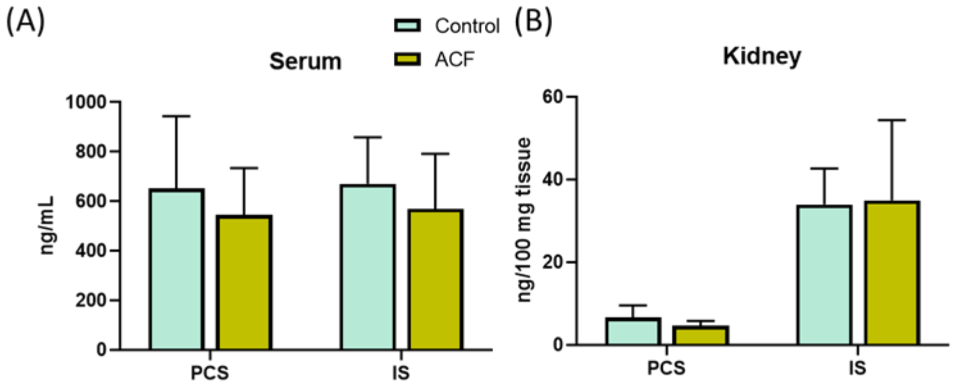

Scheme 1. Levels of p-cresol sulfate (PCS) and indoxyl sulfate (IS) in plasma (A) and kidney (B). Samples were analyzed by LC-TQ mass spectrometry in control (n = 7) and ACF rats (n = 5) after surgery. Data are reported as mean ± SEM. Differences between the two groups were determined by the Mann-Whitney U test.

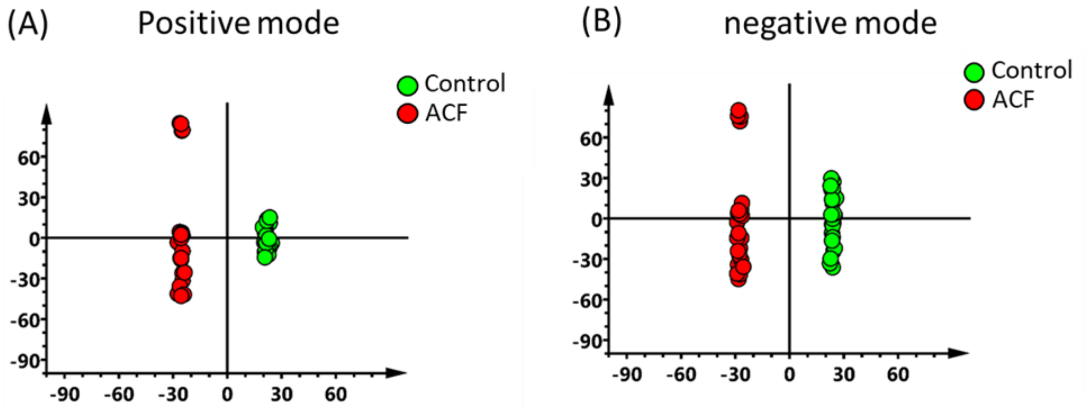

Scheme 2. Scores plot from the multivariate OPLS-DA model in positive (A) and negative mode (B). Features of the scores plot include the excellent discrimination between the ACF and control groups.

Table S1. The identified metabolites in ESI positive and ESI negative mode.

ESI positive	Metabolites	Adduct	m/z	Retention time	Identification
1	Carnitine	M+H	162.113	3.295	Lab standard
2	Acetylcarnitine	M+H	204.122	2.7604	Lab standard
3	Phosphocholine	M+H	184.074	4.7828	HMDB
4	Adenosine	M+H	268.104	3.4486	Lab standard
5	Betaine	M+H	118.087	3.6662	Lab standard
6	Taurine	M+H	126.023	4.0957	Lab standard
7	NAD	M+H	664.116	5.5022	Lab standard
8	Creatine	M+H	132.077	3.7148	Lab standard
9	Guanine	M+H	152.057	3.8644	Lab standard
10	Propionylcarnitine	M+H	218.139	2.4975	HMDB

11	Choline	M+H	104.108	2.0194	Lab standard
12	Hydroxyisovaleroyl carnitine	M+H	262.165	2.911	HMDB
13	Anserine	M+H	241.129	4.9764	Lab standard
14	Deoxycytidine	M+H	228.099	3.851	HMDB
15	3-Methylhistidine	M+H	170.092	5.0313	HMDB
16	Methylaspartic acid	M+H	148.061	4.1864	HMDB
17	Tryptophan	Fragment	188.071	3.4472	Lab standard
18	Cytosine	M+H	112.051	4.1777	HMDB
19	5'-Methylthioadenosine	M+H	298.097	2.8832	HMDB
ESI negative					
1	Inosine	M-H	267.072	3.487	Lab standard
2	AMP	M-H	346.053	5.015	Lab standard
3	Pyroglutamic acid	M-H	128.034	2.508	Lab standard
4	Xanthine	M-H	151.024	3.310	Lab standard
5	Glucuronolactone	M-H	175.022	2.600	HMDB
6	Tyrosine	M-H	180.066	3.875	Lab standard
7	Succinyladenosine	M-H	382.098	3.406	HMDB
8	Malic acid	M-H	133.013	2.673	Lab standard
9	Gluconic acid	M-H	195.049	4.057	HMDB
10	Glutamate	M-H	146.044	4.313	Lab standard
11	Uric acid	M-H	167.020	3.986	Lab standard
12	Glucose 6-phosphate	M-H	259.021	5.370	Lab standard
13	Ascorbic acid-2-sulfate	M-H	254.980	2.129	HMDB
14	Cholic acid	M-H	407.279	2.375	Lab standard
15	Glucose	M-H	179.055	4.171	Lab standard
16	S-Adenosylhomocysteine	M-H	383.113	4.893	Lab standard
17	3,4-Dihydroxybutyric acid	M-H	119.035	4.831	HMDB
18	5-Glutamyl-taurine	M-H	253.048	4.742	HMDB
19	Phosphoethanolamine	M-H	140.011	4.892	Lab standard
20	N-Acetylglutamine	M-H	187.072	2.939	HMDB
21	Tyrosol 4-sulfate	M-H	217.016	2.246	HMDB

Methods

Determine the levels of p-cresyl sulfate and indoxyl sulfate in rat plasma and kidney tissue using UPLC-triple quadrupole mass spectrometry (TQ-MS)

For the analysis of uremic toxins, 100 μL of plasma was extracted with 200 μL of acetonitrile containing 0.1% formic acid. The mixture was vortexed for 30 seconds and sonicated for 15 min on an ice bath to ensure thorough mixing. Following centrifugation at 12,000 g for 30 min at 4°C, the supernatant was transferred to another microtube. The remaining pellet was extracted again with 200 μL of 50% methanol containing 0.1% formic acid and following the same procedure. Both the methanolic supernatant and acetonitrile supernatant were combined and dried under a stream of nitrogen gas. For the analysis of kidney tissue, the protocol was the same as the hydrophilic metabolic extraction.

Prior to analysis, both plasma and tissue samples were dissolved in 200 μL of 50% acetonitrile and centrifuged at 12,000 g for 30 min. The clear supernatant was collected for UPLC-TQ-MS analysis. Tandem mass spectrometry was performed using a Xevo TQS MS system (Waters Corp., Milford, USA). Separation was achieved on a reversed-phase Acquity UPLC HSS T3 C18 column (2.1 × 100 mm, 1.8 μm) at 35 °C using eluent A (water with 0.1% formic acid) and eluent B (acetonitrile with 0.1% formic acid) in a gradient profile: 1% B for 0.5 min, increasing to 40% B within 0.5 min, and kept for 1.5 min, then increased to 99% B for 1 min, and finally re-equilibrated for 2 min at 1% B. The flow rate was 350 μL/min. MS was performed in negative-ion multi-ple-reaction-monitoring (MRM) mode. Single analyte standards were dissolved in a mixture of water/methanol 50:50 (v/v) and were then infused at a flow rate of 10 μL/min for tuning purposes. The major MS/MS fragment patterns of each analyte were determined. The optimized parameters were as follows: the capillary was set at 1500 V, the cone voltage was set at 30 V and the cone gas flow was 150 L/h, the source temperature was set at 150 °C. The desolvation gas flow was set at 1000 L/h at a temperature of 500 °C. The mixed quality control (QC) samples were analyzed during the analytical runs after every 10th sample. Data were collected and processed using Masslynx software (version 4.0).
